# Designing of the Anticancer Nanocomposite with Sustained Release Properties by Using Graphene Oxide Nanocarrier with Phenethyl Isothiocyanate as Anticancer Agent

**DOI:** 10.3390/pharmaceutics10030109

**Published:** 2018-08-01

**Authors:** Dasan Mary Jaya Seema, Bullo Saifullah, Mariadoss Selvanayagam, Sivapragasam Gothai, Mohd Zobir Hussein, Suresh Kumar Subbiah, Norhaizan Mohd Esa, Palanisamy Arulselvan

**Affiliations:** 1 Department of Advanced Zoology and Biotechnology, Loyola Institute of Frontier Energy (LIFE), Loyola College, Chennai 600034, India; dmjseema2k@gmail.com (D.M.J.S.); selvam.mariadoss@gmail.com (M.S.); 2 Material Synthesis and characterization laboratory, Institute of Advanced Technology (ITMA), Universiti Putra Malaysia, Serdang 43400, Malaysia; bullosaif1@gmail.com (B.S.); mzobir@upm.edu.my (M.Z.H.); 3 Laboratory of Vaccines and Immunotherapeutics, Institute of Bioscience, Universiti Putra Malaysia, Serdang 43400, Malaysia; gothailogishkumar@gmail.com; 4 Henan-Macquarie Universities Joint Center for Biomedical Innovation, School of life Sciences, University of Henan Jin Ming Avenue, Kaifeng 475004, China; 5 Loyola-ICAM college of engineering and Technology (LICET), Loyola Campus, Chennai 600034, India; 6 Department of Medical Microbiology and Parasitology, Faculty of Medicine and Health Sciences, Universiti Putra Malaysia, Serdang 43400, Malaysia; sureshkudsc@gmail.com; 7 Department of Nutrition and Dietetics, Faculty of Medicine and Health Sciences, Universiti Putra Malaysia, Serdang 43400, Malaysia; nhaizan@upm.edu.my; 8 Muthayammal Centre for Advanced Research, Muthayammal College of Arts and Science, Rasipuram, Namakkal, Tamilnadu 637408, India; 9 Scigen Research and Innovation, Periyar Technology Business Incubator, Periyar Nagar, Thanjavur, Tamilnadu 613403, India

**Keywords:** graphene oxide, anticancer, phenyisothiocyanate, nanocomposite, nano-carrier

## Abstract

In this study anticancer nanocomposite was designed using graphene oxide (GO) as nanocarrier and Phenethyl isothiocyanate (PEITC) as anticancer agent. The designed formulation was characterized in detailed with XRD, Raman, UV/Vis, FTIR, DLS and TEM etc. The designed anticancer nanocomposite showed much better anticancer activity against liver cancer HepG2 cells compared to the free drug PEITC and was also found to be nontoxic to the normal 3T3 cells. In vitro release of the drug from the anticancer nanocomposite formulation was found to be sustained in human body simulated phosphate buffer saline (PBS) solution of pH 7.4 (blood pH) and pH 4.8 (intracellular lysosomal pH). This study suggests that GO could be developed as an efficient drug carrier to conjugate with PEITC for pharmaceutical applications in cancer chemotherapies.

## 1. Introduction

The application of nanotechnology for biomedical application is one of the most vital advancement in the field of science in this century. This technology not only advanced the medical diagnostics, drug delivery and drug manufacturing but also made possible simultaneous detection and cure (i.e., theranostic) of wide range of diseases [1,2,3,4]. In nanomedicine the area of drug delivery research is a subject of rapid growth and advancement due its significant role in the improvement of therapeutic efficacy of the drugs, minimization of the adverse effects of the drugs and enhancement in the bioavailability of the drugs etc. [5,6,7]. Many nanosized materials namely liposomes, micelles, dendrimers, carbon nanotubes, polymers, inorganic metallic nanolayers and graphene oxides have been explored for the designing of the nanocarriers for different drugs [8,9,10,11,12].

Graphene oxide (GO) is a promising functional nanobiomaterial that is widely applied in drug delivery, biosensing, energy storage devices (supercapacitor and batteries), electronics, photocatalysis and in biomedicine [13,14,15]. GO and its modified forms have been getting more and more attention in scientific community because of its multifunctional surface and multidimensional applications in different fields. In biomedicine GO is mainly used for drug delivery, bioimaging, cancer therapy and in biosensors due to its biocompatibility and physico-chemical properties. GO has unique structure with graphene basal plane functionalized with carboxylic (COOH), hydroxyl (OH), epoxides groups etc. These functional groups enable GO for further functionalization, conjugation and/or immobilization of other nanoparticles and loading of drugs/biomolecules (RNA/DNA etc) on its surface [15].

Recently GO has been widely applied in drug delivery especially for cancer therapy and have got potential to overcome the shortcomings of current cancer chemotherapy. The drugs can easily be loaded on GO via the pi-pi stacking interaction and hydrogen bonding [15,16,17,18]. The GO and gold (Au) rod hybrid nanocomposites loaded with anticancer drugs have been reported to possess excellent drug release, improved photothermal and photo acoustic effect in killing of cancer cells [16]. Furthermore, GO also interacts with near infrared (NIR) and produces heat inside the cancer cells [15,19]. This heat generation property of GO upon NIR can be exploited in the GO nanocomposites with radionuclide which can release X-ray upon heating, can potentially be applied in cancer therapy and due to the multiple beneficial effects, the efficient eradication of tumour cells is possible [20]. Graphene oxide alone has also been utilized for delivering anticancer drugs such as Chlorogenic acid, Gallic acid and Doxorubicin etc. [3,18,21,22].

Isothiocyanates (ITCs) are the Sulfur containing natural products formed by the reaction of glucosinolates with myrosinase, an enzyme released by the disruption of plant tissues. This myrosinase-glucosinolate system is available in plant family Brassicaceae, such as broccoli, cauliflower, cabbage and mustard. The ITCs have been reported to possess anticancer and antimicrobial properties [23,24,25,26]. Phenethyl isothiocyanate (PEITC) has been reported to have good anticancer activity in both in vitro and in vivo models [27,28,29]. In this study we designed anticancer nanocomposite by loading PEITC on graphene oxide and characterized in detail and evaluated for anticancer properties.

Several studies reported that, PEITC could potentially induce cell cycle arrest and apoptotic cell death in several cancer cell lines [30]. Inhibition of cell viability with same IC50 values has been achieved for both MCF-7 and MCF–12A breast cancer cell lines when treated with PEITC [31]. In human prostate cancer DU 145 Cells, PEITCs were known to induce apoptosis via the mitochondrial apoptosis pathway [32]. Hyung Shim et al., suggested PEITC as a dietary compound for cervical cancer patients due to its promising cytotoxic effects towards human cervical cancer cells [33]. PEITC has also been found to induce cytotoxicity in dose and time dependent manner by triggering apoptosis in HepG2 human liver carcinoma cells [34]. A comparative study on HepG2 and B16F10 cell lines were also reported with the maximum percent of PEITC induced inhibition on HepG2 cells [35].

In recent years, more promising and interesting results are reported, after conjugating anticancer drugs with various pharmacophoric units. Yan and coworkers encapsulated PEITC and CDDP in approximately 130 nm liposomes and observed antiproliferative effects as well as an immense decrease in tumor and reduced symptoms in lung cancer [36]. Graphene oxide-iron oxide (GO-IO) nanocomposites were prepared and studied on breast cancer 4T1 cells [37]. Highly reduced graphene oxide with silver decorated nanocomposites showed potent anticancer properties on A549 human lung cancer cells compared to that of the free drug used [38]. Highly encouraging anticancer activity was observed when HepG2 cells were treated with Graphene oxide-gallic acid (GOGA) nanocomposite [39]. Recently, graphene oxide- chlorogenic acid nanocomposite that was synthesized exhibited potential cytotoxic effects on HepG2 cell line and negligible toxicity towards the normal cell line used [40]. Above studies suggest that, much more can be done in order to further exploit the biological applications of graphene oxide nanocomposites in understanding chemotherapeutic efficacy of potentially active PEITC on various cancers. Hence, we believed that combination of GO and PEITC therefore will open door to further understand more about the potentially active PEITC and may facilitate new approaches for drug development. Keeping this in view, we designed, synthesized and characterized the graphene oxide-Phenethyl isothiocyanate (GO-PEITC) nanocomposite and investigated it’s the cytotoxic studies on HepG2 liver cancer cells and normal fibroblast 3T3 cells.

## 2. Materials and Methods

### 2.1. Materials

Graphite flakes (109 meshes), sulphuric acid (H_2_SO_4_ 98%), phosphoric acid (H_3_PO_4_), potassium permanganate (KMnO_4_), hydrogen peroxide, and phosphate buffered saline (PBS) purchased from Sigma Aldrich (St Louis, MO, USA) and utilized without further purification. Phenethyl isothiocyanate (PEITC) Sigma, St Louis, MO, USA, diethyl ether, sodium hydroxide, hydrochloric acid (HCl, 37%), Ethyl alcohol (99.7% *v*/*v*) were bought from Friedemann Schmidt (Parkwood, WA, USA). 3-(4,5-di-methyl-2-thiazolyl)-2,5-diphenyl-2H-tetrazolium bromide (MTT), heat-inactivated fetal bovine serum (FBS), Dulbecco’s modified eagle medium (DMEM), antibiotics penicillin-streptomycin and deionized water was used in all experiments.

### 2.2. Cell Culture and Maintenance

Human hepatocarcinoma (HepG–2) and 3T3 (normal standard fibroblast) cell lines were cultured under standard cell culture conditions (37 °C in a humidified atmosphere of 95% room air/5% CO_2_) in Dulbecco’s modified Eagle’s medium (DMEM) supplemented with 10% heat inactivated fetal bovine serum (FBS), and a 1% mixture of penicillin/streptomycin. Cells were sub cultured in 75 cm^2^ culture flasks or in appropriate plates and used for seeding and treatment after reaching approximately 80% confluence.

### 2.3. Synthesis of Graphene Oxide

Graphene oxide (GO) was synthesized by improved method. In brief concentrated H_2_SO_4_ (360 mL) was mixed with 40 mL concentrated H_3_PO_4_ and was added to mixture of 3 g graphite powder and 18 g KMnO_4_. The solution was kept on stirring at 50 °C for 12 h. After that resultant suspension was poured on the 400 g ice cubes containing 3 mL of hydrogen peroxide and then final solution was washed with 200 mL deionized water, 200 mL HCl and 200 mL ethanol. Finally, sample was coagulated with diethyl ether and then dried at 40 °C [41].

### 2.4. Drug loading on GO

In brief 2 mL of PEITC was dissolved in 100 mL of ethanol and to this solution 0.5 g of GO was added and solution was stirred for 24 h. After that sample was centrifuged, washed thoroughly and dried in oven at 40 °C and subjected to characterization.

### 2.5. Physicochemical Characterization

X-ray diffraction (XRD) patterns were recorded using condition CuKα radiation (λ = 1.5418 Å) at 30 kV and 30 mA with XRD-6000 Diffractometer, Shimadzu, Tokyo, Japan). A Perkin Elmer ultraviolet-visible spectrophotometer model lambda 35 was utilized for the quantification of drug loading and in vitro release properties. High resolution transmission electron microscope (HR-TEM model Tecnai G2 (FEI Company, Hillsboro, OR, USA) was used for the surface and morphological properties. For the Raman analysis Raman spectrometer (model Alpha 300R Witec, Ulm, Germany) with an excitation wavelength at 532 nm was utilized. Functional groups bands were recorded in the range of 500–4000 cm^−1^ analyzed by Fourier Transformed Infrared (FTIR) spectrometer Perkin-Elmer 100 series spectrophotometer (Waltham, MA, USA) by a direct sample method.

### 2.6. Determination of Anticancer Activity

#### Cytotoxicity Assay

Cytotoxicity of native PEITC and PEITC-loaded GO (GO-PEITC) on HepG2 cell line was analyzed by the MTT calorimetric assay. In brief, all the normal and HepG2 cell lines were seeded in 96 well plates at the density of 1 × 10^4^ cells per well and kept in an incubator for up to 24 h for acclimatization. After 24 h, cells were treated with various concentrations (0–10 μg/mL) of native PEITC, GO and GO-PEITC. Cells were incubated in a CO_2_ incubator at 37 °C for 72 h. After 48 h of incubation, medium was removed and 100 μL of fresh medium was added along with (5 mg mL^−1^) MTT solutions and incubated for another 4 h in a CO_2_ incubator. The extent of cell viability was determined by the conversion of yellow MTT into purple formazan by the living cells. After the medium was aspirated, the formed formazan crystals were dissolved in 200 μL of dimethyl sulfoxide (DMSO) and its absorbance was measured at 540 nm using a microplate reader. All assays were done in triplicate and the cytotoxicity results were expressed as the percentage of cell viability with respect to control cells.

Cytotoxicity (%) = [100 × (Absorbance of untreated group − Absorbance of treated group)/Absorbance of untreated group].

## 3. Results

### 3.1. Powder X-ray Diffraction (PXRD)

**Figure 1:**
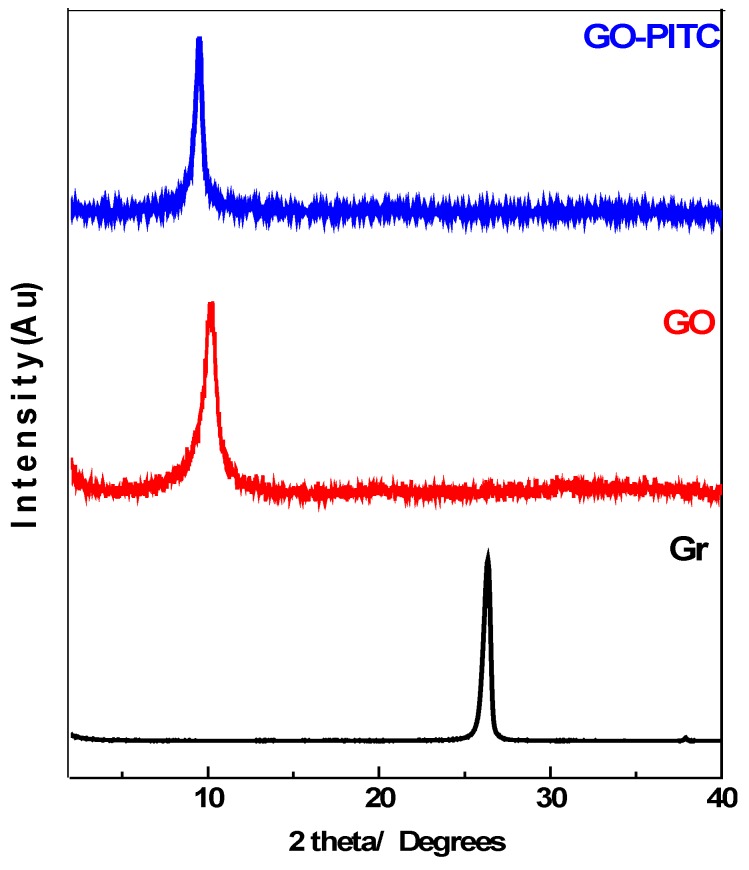
X-ray diffraction patterns of Graphite (Gr), graphene oxide (GO) and the GO-PEITC nanocomposite.

Figure 1, shows the PXRD patterns of graphite (Gr), graphene oxide (GO) and graphene oxide-phenyl isothiocyante nanocomposite (GO-PEITC). Graphite (Gr) showed the sharp and strong characteristic peak at 2θ degree 26.1° corresponding to diffraction of (002) plane with basal spacing of about 3.4 Å [18]. In the PXRD diffraction patterns of GO, the Gr peak disappeared and new characteristic GO peak appeared at about 2θ degree 10.3° with basal spacing of 8.5 Å. The increased basal spacing from 3.4 Å to 8.5 Å is attributed to the insertion of oxygenated functional groups namely carboxylic acid, epoxides and hydroxyl groups between the GO planes [18,42,43]. The disappearance of Gr peak from 26.1° and appearance characteristic GO peak at about 10.20° degrees with increased basal spacing strongly indicates the successful formation of GO [17,21]. In the nanocomposite GO-PEITC, the GO peak has been slightly shifted to lower 2θ degree i.e., 9.5° with slight increase in basal spacing which can be attributed to the loading of PEITC on GO.

### 3.2. Infrared Spectroscopy

**Figure 2:**
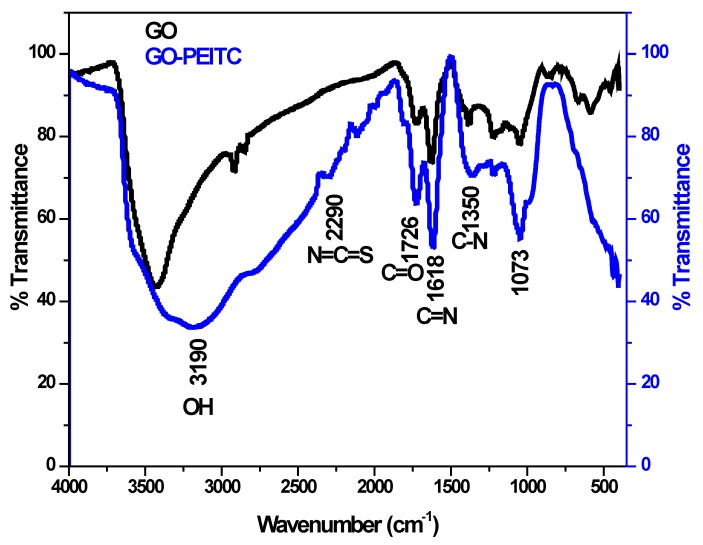
Fourier transformed infrared spectra of GO and the nanocomposite Go-PEITC.

Figure 2 shows the Fourier transformed infrared spectra of GO (black colour) and of the nanocomposite GO-PEITC (blue colour). The infrared spectrum of GO showed the characteristic functional group bands corresponding to hydroxyl group at about 3400 cm^−1^, carbonyl group (C=O) 1726 cm^−1^ and alkoxide group (C–O) 1072 cm^−1^ respectively [18,21]. The nanocomposite GO–PEITC showed the infrared absorption bands of GO as well as the characteristic functional group bands of PEITC namely N=C=S at 2290 cm^−1^, C=N at 1618 cm^−1^ and C–N band at 1350 cm^−1^ as reported in literature [33,34,44]. The presence of GO and PEITC bands in the nanocomposite GO-PEITC strongly suggest the successful formation of the nanocomposite.

### 3.3. Schematic Representation of the Structures

**Figure 3:**
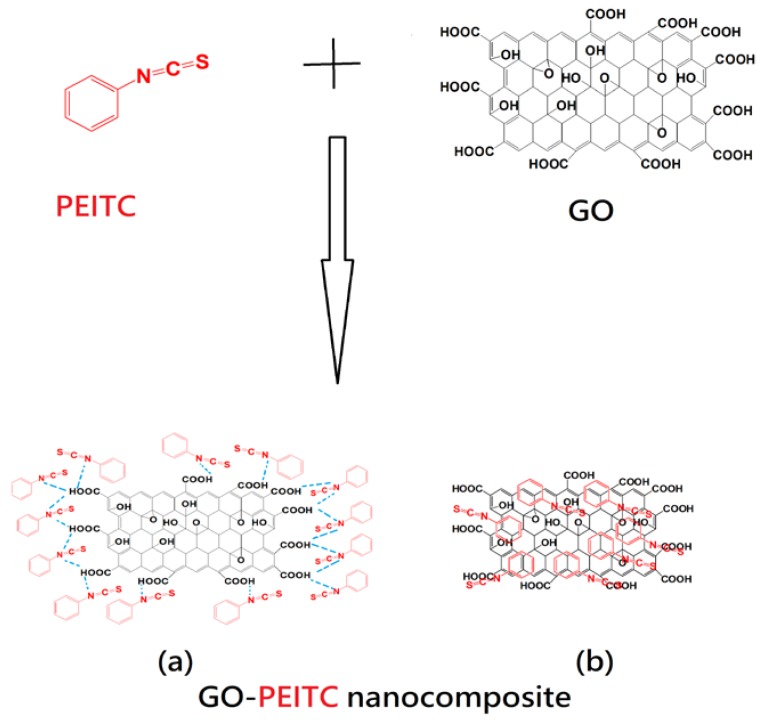
Structure of phenethyl isothiocyanate (PEITC), GO and of the nanocomposite GO-PEITC: (**a**) shows the hydrogen bonding and (**b**) shows the pi-pi stacking between GO and PEITC.

Figure 3 shows the structures of phenyl isothiocynate PEITC, GO and the final nanocomposite GO-PEITC. The drug can possibly interact and loaded on GO by hydrogen bonding (a) as well as pi-pi stacking (b). Here we have shown both phenomena, hydrogen bonding and pi-pi stacking separately for clarity. In the structure of the nanocomposite GO-PEITC, PEITC is represented in red colour, GO is represented in black colour and hydrogen bonding is represented in blue colour.

### 3.4. Raman Spectroscopic Analysis

**Figure 4:**
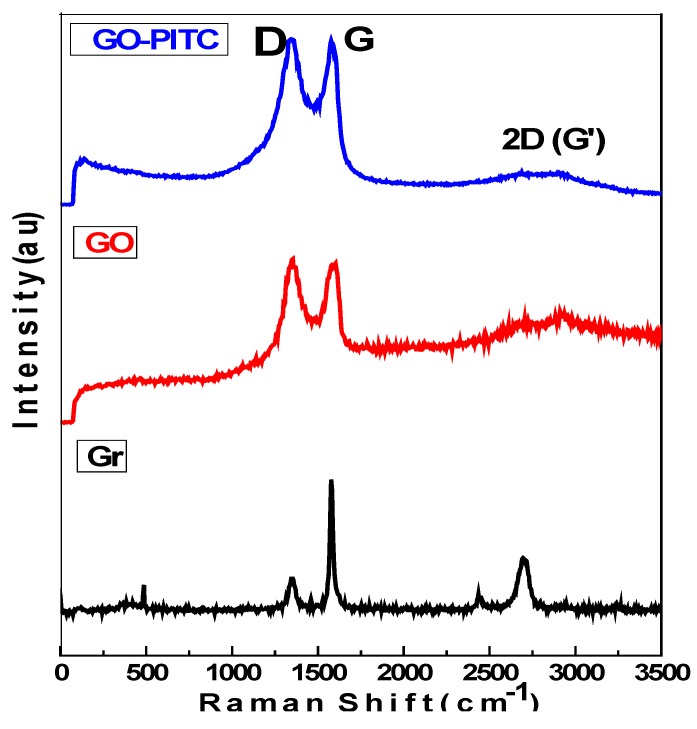
Raman spectra of Gr, GO and the nanocomposite GO-PEITC.

Raman spectroscopy was used for the determination of structural changes induced during chemical reactions of the resulting materials namely GO and nanocomposite GO-PEITC. Figure 4 shows the Raman spectra of graphite (Gr), GO and of the nanocomposite GO-PEITC. The starting material Gr showed two main peaks i.e., d-band due to the disorder induced mode and the G-band (graphitic like mode) present at 1356 and 1581 cm^−1^ respectively. In Raman spectroscopy D-band represents the non-crystalline quality of the carbon associated with defects and disorder of the material under consideration, whereas the G-band is associated with high degree of ordering of the crystalline graphitic structure [18,30,45,46]. As it can be seen in the Raman spectrum of graphite (Gr) (Figure 4.) intensity of G-band is very high due to the high order of the crystallinity whereas d-band intensity is very low representing lesser defects present in Gr. In the Raman analysis of both GO and the nanocomposite GO-PEITC (Figure 4) the intensity of G-band is considerably decreased whereas the intensity of d-band is increased at the same time. This suggest the high degree of disordering inducted by the oxidative exfoliation of GO and by the loading of the PEITC on GO.

### 3.5. Transmission Electronic Microscopic (TEM) Analysis

**Figure 5:**
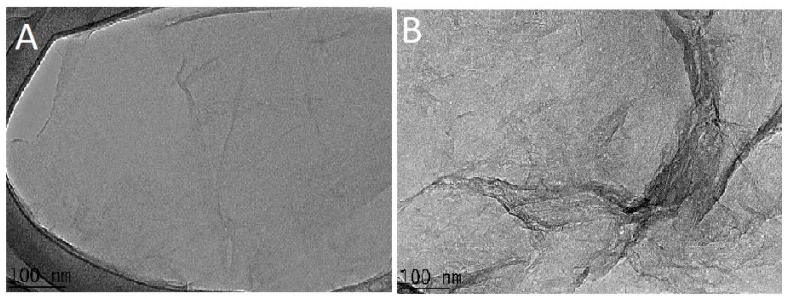
(**A**) HR-TEM image of GO and (**B**) the nanocomposite GO-PEITC.

TEM micrographs of GO and the nanocomposite GO-PEITC are presented in the Figure 5. The structural features and morphology of GO and GO-PEITC were analyzed by high resolution transmission electron microscopy (HR-TEM). The starting material graphite (Gr) exhibits large number of stacked graphene sheets, as we reported previously [18,21]. Unlike Gr the TEM image shows the smooth regular structure of GO (Figure 5A) which indicates the successful conversion of Gr into GO [30]. The Figure 5B represents the HR-TEM image of the nanocomposite GO-PEITC which is slightly different in appearance with some of the defects on the surface which may possibly be attributed to the loading of PEITC on the surface of GO.

### 3.6. Particle Size Analysis

**Figure 6:**
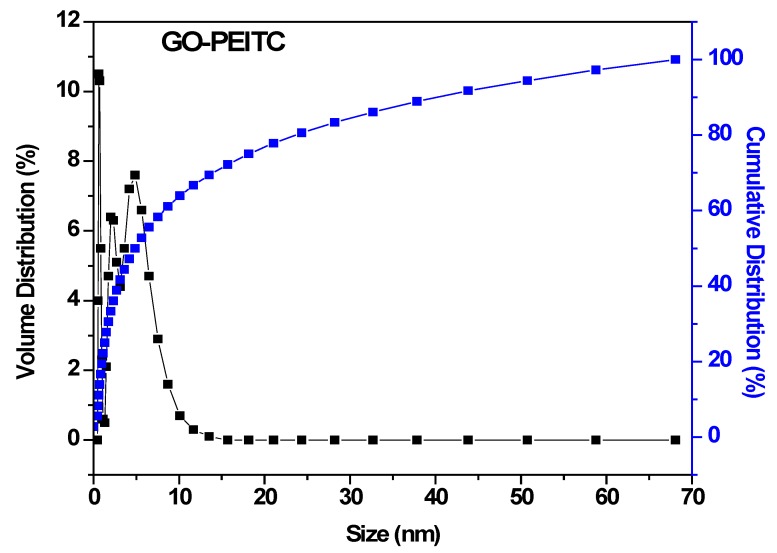
Hydrodynamic size of the nanocomposite GO-PEITC determined by DLS.

Particle size of the nanocomposite GO-PEITC was determined by dynamic light scattering (DLS) analysis using Zetasizer technique. The sample was dispersed in water followed by sonication for 20 min and then analysed with Zetasizer. The GO-PEITC was found to have very narrow size distribution ranging from 0 to 15 nm (Figure 6). The Cumulative distribution frequency revealed that 60% of the particles were of the size below 6 nm.

### 3.7. Release Studies

**Figure 7:**
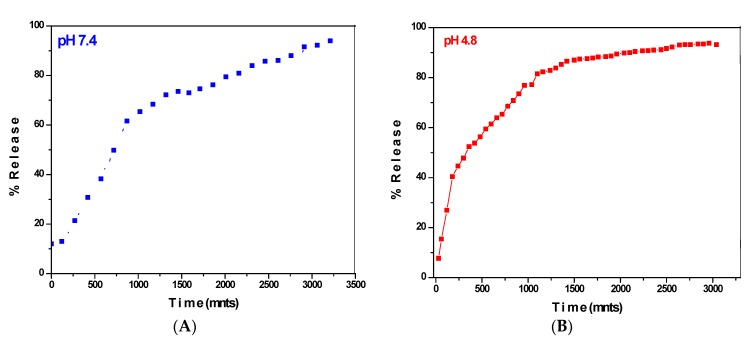
In vitro release profile of PEITC from the nanocomposite GO-PEITC in human body simulated PBS solution of pH 7.4 (**A**) and pH 4.8 (**B**) in vitro cytotoxicity analysis.

Figure 7, represents the in vitro release of PEITC drug under human body simulated phosphate buffer saline (PBS) solutions of pH 7.4 (blood pH) and pH 4.8 (intracellular lysosomal pH). Figure 7A shows the release profile of the PEITC from the nanocomposite GO-PEITC in PBS solution of pH 7.4. It can been observed that initial 70% of drug release took about 1500 min and remaining 30% of release took about additional 1500 min for the sustained release. The PEITC took 3000 min overall, for 100% release. Figure 7B shows the release profile of PEITC in PBS solution of pH 4.8. Initially there is relatively faster release which took about 500 min for 50% release and remaining drug release took additional 1000 min. The overall release took 1500 min to be released completely. So the in vitro release of the PEITC from the nanocomposite GO-PEITC is sustained under both physiological conditions of pH 7.4 and pH 4.8. However, the release of the drug is more sustained in PB solution of pH 7.4 (3000 min) compared to the release profile in PBS solution of pH 4.8 (1500 min).

**Figure 8:**
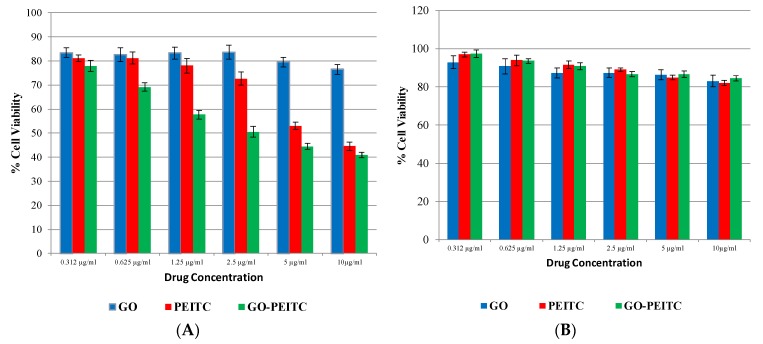
Effects of GO, PEITC and PEITC-loaded GO (GO-PEITC) (0–10 µg/mL) on viability of (**A**) HepG2 and (**B**) 3T3 cell line and the cell viability was measured by MTT assay (72 h). Data represented as mean ± SE of three independent experiments made in three replicates.

The MTT (3-(4,5-dimethylthiazolyl-2)-2,5-diphenyltetrazolium bromide) assay measures the cell proliferation rate and conversely, the reduction in cell viability when metabolic events lead to apoptosis [37]. The yellow compound MTT is reduced by mitochondrial dehydrogenases to the water insoluble purple formazan compound, depending on the viability of the cells. Therefore, the efficacy of GO-PEITC nanocomposite in suppressing the growth of HepG2 cell line was assessed using colorimetric assay (MTT assay). The 3T3 normal fibroblast cells were employed as normal control to ensure the nontoxic nature of nanocomposite and also nano-carrier alone. The cytotoxicity effect on HepG2 cell line was studied at different concentration (0 μg/mL–10 μg/mL). As can be seen from Figure 8A GO-PEITC demonstrated better anti-proliferative activity than pure compound, PEITC. The IC_50_ value was found approximately to be 7.5 μg/mL for the pure compound PEITC. GO-PEITC was found to be much more effective inhibitor of HepG2 cells growth with IC_50_ value of about 2.5 μg/mL. Observation from IC_50_ values indicated that enhanced anti-proliferating efficiency by GO-PEITC (based on the drug loading concentration) than that of pure compound, PEITC. This shows that the introduction of GO into PEITC improved the efficacy of PEITC in HepG2 cells. It is apparent from Figure 8B that pure compound, PEITC and nanocomposite GO-PEITC did not affect normal fibroblast cell viability in the tested range, as the survival was consistently greater than 80% or similar to control. In this case, it can be indicated that GO-PEITC nanocomposite possessed beneficial anticancer activities compared to that of the pure compound, PEITC and demonstrated selectivity between cancerous and normal fibroblast cells.

## 4. Conclusions

In this study, anticancer nanocomposite was designed using Phenethyl isothiocyanate (PEITC) as anticancer agent and graphene oxide (GO) as effective nanocarrier. The designed anticancer nanocomposite GO-PEITC was found to release the drug PEITC in sustained manner in blood and intercellular lysosomal condition of PBS pH 7.4 and pH 4.8, respectively. The designed anticancer nanocomposite showed better anticancer activity (i.e., IC_50_ value of 2.5 μg/mL) compared to pure compound, PEITC (i.e., IC_50_ value of 7.5 μg/mL). In addition, the nanocomposite GO-PEITC was found to be highly biocompatible with normal cells.
